# Oxygen release from metal oxide for repeated hydrogen regeneration by proton irradiation with polyvinylpyrrolidone

**DOI:** 10.1039/c8ra02577k

**Published:** 2018-05-22

**Authors:** Keumyoung Seo, Taekyung Lim, Sang-Mi Jeong, Sanghyun Ju

**Affiliations:** Department of Physics, Kyonggi University Suwon Gyeonggi-Do 443-760 Republic of Korea shju@kgu.ac.kr

## Abstract

In this study, we investigated the reduction of a 3D microporous NiO_*x*_ structure, used as a metal oxide catalyst, by proton irradiation with polyvinylpyrrolidone (PVP) for hydrogen regeneration. In general, the reduction process for hydrogen regeneration requires high temperatures (1000–4000 °C) to release saturated oxygen from the metal oxide catalyst. Proton irradiation with PVP could regenerate abundant oxygen vacancies by releasing the oxygen attached to NiO_*x*_ at room temperature. The 3D microporous NiO_*x*_ structure provided the maximum hydrogen generation rate of ∼4.2 μmol min^−1^ g^−1^ with the total amount of generated hydrogen being ∼460 μmol g^−1^ even in the repetitive thermochemical cycle; these results are similar to the initial hydrogen generation data. Therefore, continuous regeneration of hydrogen from the oxygen-reduced 3D microporous NiO_*x*_ structure was possible. It is expected that the high thermal energy, which is the major problem associated with hydrogen regeneration through the conventional heat treatment method, would be resolved in future using such a method.

## Abstract 2

The reduction of a 3D microporous NiO_*x*_ structure, used as a metal oxide catalyst, was performed by proton irradiation with polyvinylpyrrolidone (PVP) for hydrogen regeneration.

## Graphical abstract

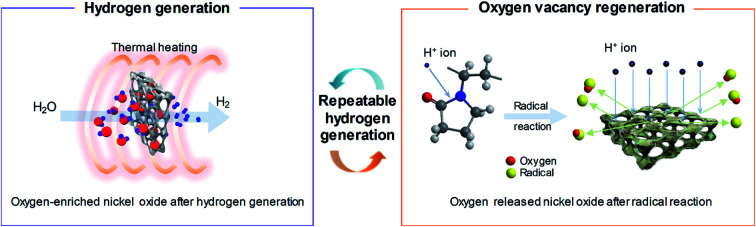

## 1. Introduction

Hydrogen has been actively studied as a renewable energy source that can replace fossil fuels, which suffer from the problems of rapidly depleting resources and the environmental pollution associated with their use.^1,2^ Hydrogen is a clean energy source that can be obtained indefinitely not only from the existing fossil fuels, but also from water and other natural organisms. However, hydrogen energy systems, which are currently being commercially used, produce hydrogen by decomposing mainly natural gas or methane gas; thus, the use of fossil fuel is inevitable. To establish hydrogen energy as a renewable energy, it is essential to study environment-friendly and cost-effective processes for the generation of hydrogen.

One of the representative environment-friendly hydrogen generation methods in the current stage of technological advancement is the water splitting method using alternative energy sources (solar, wind, and the latent heat of a power plant), based on photocatalysts, photosynthesis and microorganisms, and low-temperature thermochemical cycles.^2^ Among these methods, is the water splitting method based on a low-temperature thermochemical cycle, in which redox-accessible medium materials in a closed cycle configuration decompose water at a temperature of 1000 °C or less through a multiple-step chemical reaction. Theoretically, such a method can achieve a thermal efficiency of 80% or more, and has the advantage of using thermal energy sources like solar energy and the latent heat of a power plant.^3,4^ Since the first study on thermochemical water splitting conducted by Funk and Reinstrom in 1964, over 200 water decomposition thermochemical cycles have been proposed thus far. However, for a simple two-step thermochemical cycle, the use of the water-splitting method is limited when the redox reaction of the medium occurs at a reaction temperature as low as 1000 °C or less.^5^ Therefore, it is necessary to study the methods in which metal oxides are reduced using a lower energy because the reduction energy required for reduction of a metal oxide for hydrogen regeneration is higher than the oxidation energy required for hydrogen generation. Although studies on various metal oxide reduction methods are in progress, further research is required on metal oxide reduction methods that use lower energy.^6^

In this study, repeated regeneration of hydrogen on a 3D microporous NiO_*x*_ structure with large specific surface area used as a metal oxide catalyst was carried out by proton irradiation with polyvinylpyrrolidone (PVP). After coating the PVP as a reduction catalyst onto the oxidized NiO_*x*_ after hydrogen generation, the oxygen-rich NiO_*x*_ could be effectively reduced by using the proton irradiation method. In addition, the changes in the pore size, specific surface area, and residual oxygen content of the 3D microporous NiO_*x*_ structures were investigated before and after hydrogen generation as well as after proton irradiation with PVP.

## 2. Methods

### 2.1 Thermochemical water splitting for hydrogen generation

A 3D microporous NiO_*x*_ structure (110 ppi, 350 g m^−2^) was used as a catalyst for hydrogen generation. The size and thickness of the square 3D microporous NiO_*x*_ structure were 25 mm^2^ and 1.6 mm, respectively. After cleaning the 3D microporous NiO_*x*_ structure ultrasonically using acetone, IPA, and deionized (DI) water for 10 min, respectively, it was placed in a dry oven and completely dried at 100 °C for 30 min. Subsequently, ten-3D microporous NiO_*x*_ structures were placed in a thermal reaction chamber. After the temperature of the reaction chamber was raised to 800 °C, 100 sccm of H_2_O vapor, which is a precursor used for hydrogen generation, and 200 sccm of He gas (99.999%) as a carrier gas were supplied into the chamber. DI water was mixed with a He carrier gas in the form of water vapor and flowed into the reaction chamber through a gas line maintained at 160 °C. During the process, H_2_O vapor, which did not react with the 3D microporous NiO_*x*_ structure, was collected in a cold trap. Thereafter, 4 cm^3^ of the produced hydrogen gas was collected every 4.3 min using a gas chromatograph (Agilent 7890B) and analyzed. At this time, the hydrogen generation and regeneration processes were performed once to maintain stable generation of hydrogen even during repetitive hydrogen generation. For accurate measurement, the calibration baseline was secured by a He and H_2_ mixed standard gas (0.02, 1.03, and 3.12%), and the amount of hydrogen actually produced was analyzed based on this.

### 2.2 Oxygen vacancy regeneration *via* proton irradiation with PVP

A PVP (Sigma-Aldrich; *M*_w_ ∼ 55 000) solution was used as a reducing catalyst to reduce oxygen from the 3D microporous NiO_*x*_ structure after hydrogen generation. PVP and *N*-methyl-2-pyrrolidone (NMP, DAEJUNG, 99.5%) were mixed in a ratio of 1 : 5 and stirred for 12 h in an agitator until complete dissolution. To coat the surface of the 3D NiO_*x*_ structure with the PVP solution, the structure was dipped into the PVP solution, removed, and completely dried in air. The coating thickness of PVP was ∼30 nm. The proton irradiation process for hydrogen regeneration was performed by injecting H^+^ ions under vacuum (7 × 10^−6^ torr) using a Korea Multipurpose Accelerator Complex (KOMAC) gaseous ion beam facility. Accelerated ions with a potential difference of 50 keV were injected into the 3D microporous NiO_*x*_ structure, and the treated ion dose was 1 × 10^15^ ions per cm^2^. Note that both sides of the 3D NiO_*x*_ structure were treated. In order to remove PVP from the surface of the 3D microporous NiO_*x*_ structure after proton irradiation, it was sonicated in NMP, IPA, and DI water for 30 min, respectively, followed by dehydration in a thermal oven at 90 °C for 30 min and air drying. Subsequently, the amount of generated hydrogen was measured after loading the 3D microporous NiO_*x*_ structure, which was treated by proton irradiation with PVP, in the reaction chamber for hydrogen regeneration. The pore structure of the 3D microporous NiO_*x*_ structure before and after hydrogen generation and regeneration was analyzed by field-emission scanning electron microscopy (FE-SEM), in addition to X-ray powder diffraction (XRD) for structural analysis, Brunauer–Emmett–Teller (BET) analysis of the reaction surface area, and thermogravimetric analysis (TGA) and X-ray photoelectron spectroscopy (XPS) for surface component analysis.

## 3. Results and discussion

**Fig. 1:**
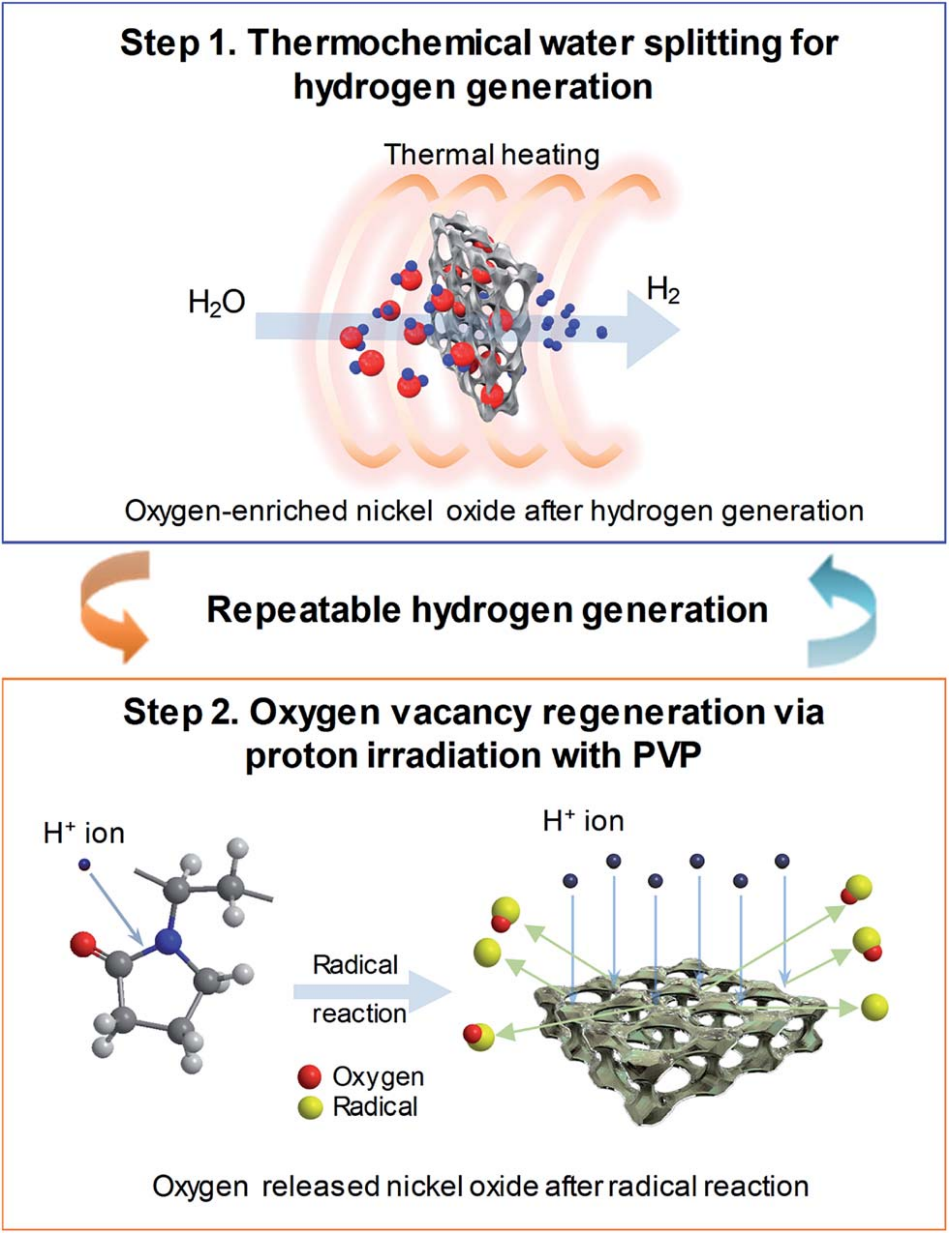
Schematic diagram of the hydrogen generation process following two-step thermochemical cycles. (Step 1) Hydrogen generation on 3D microporous NiO_*x*_ structures with thermal heating at 800 °C and (Step 2) oxygen vacancy regeneration on 3D microporous NiO_*x*_ structures for oxygen reduction using proton irradiation with PVP.

Fig. 1 shows the schematic of the water splitting method using the 3D microporous NiO_*x*_ structure with a large specific surface area as well as the reduction mechanism scheme of the NiO_*x*_ structure by proton irradiation with PVP. The principle of H_2_ formation from H_2_O through the bonding/dissociation of a metal and oxygen in a metal oxide material follows the oxidation step M_*x*_O_*y*−*z*_ + *z*H_2_O → M_*x*_O_*y*_ + *z*H_2_. Thus, when 800 °C of heat is applied to the 3D microporous NiO_*x*_ structure, nickel reacts with water vapor, resulting in its oxidation into NiO_*x*_, and produces hydrogen. The utilization of the oxygen-rich NiO_*x*_ for hydrogen regeneration after hydrogen generation involves a highly endothermic reduction step (M_*x*_O_*y*_ → M_*x*_O_*y*−*z*_ + 1/2·*z*O_2_) in the two-step water splitting process using the metal oxide as a redox pair, which requires a higher reaction temperature than that required for the slightly exothermic oxidation step (M_*x*_O_*y*−*z*_ + *z*H_2_O → M_*x*_O_*y*_ + *z*H_2_).^7–9^ This study was aimed at introducing a chemical reduction step for the metal oxide to enable continuous hydrogen generation at room temperature, as shown in the reaction NiO_*x*_ + 2*y*H^+^ → NiO_*x*−*y*_ + *y*H_2_O, as well as physical reduction of nickel oxide by irradiation with H^+^ ions using an ion beam apparatus instead of high-temperature (>1000 °C) thermal energy conventionally used in the reduction step. After the surface of the NiO_*x*_ structure oxidized during hydrogen generation was coated with the PVP solution as a reducing medium, it was irradiated with the protons generated by the ion beam device. As a result, the proton chemically reacts with the oxygen released from the NiO_*x*_ structure to produce H_2_, and the oxidized NiO_*x*_ was rapidly reduced at room temperature. The low-temperature reduction step using proton irradiation with PVP can prevent the deactivation of the repeated cyclic reaction of the metal oxide by sintering the metal oxide as in a conventional high-temperature reduction step. As a result, a similar amount of hydrogen can be produced from multiple redox reactions.

**Fig. 2:**
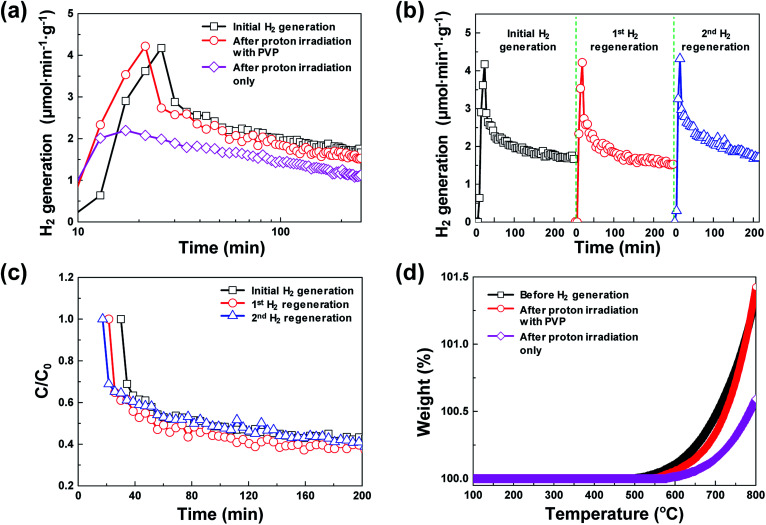
(a) Comparison of hydrogen generation characteristics of initial hydrogen generation after proton irradiation only and after proton irradiation with PVP. (b) Hydrogen generation characteristics in three repeatable hydrogen generation cycles after oxygen reduction process using proton irradiation with PVP (regeneration process). (c) Time profiles of hydrogen degradation (*C*/*C*_0_) during three repeatable hydrogen generation cycles. (d) Thermogravimetric analysis curves of 3D microporous NiO_*x*_ structures before hydrogen generation, after proton irradiation with PVP, and after proton irradiation only.

Fig. 2(a) shows a comparison between the amounts of regenerated hydrogen obtained by applying two hydrogen regeneration methods, proton irradiation only and proton irradiation with PVP, with respect to the initial hydrogen generation. The temperature required for hydrogen generation was 800 °C. For NiO_*x*_ reduced by only proton irradiation without using PVP as a reducing catalyst during hydrogen regeneration, the maximum hydrogen generation rate was ∼2.2 μmol min^−1^ g^−1^, and the total amount of generated hydrogen was ∼300 μmol g^−1^. On the other hand, in the case of NiO_*x*_ reduced by proton irradiation with PVP, the maximum hydrogen generation rate was ∼4.2 μmol min^−1^ g^−1^, and the total amount of generated hydrogen was ∼460 μmol g^−1^, which were nearly the same as that obtained in the initial hydrogen generation. Note that the maximum hydrogen generation rate and amounts of hydrogen generated from previously reported metal oxide materials (FeAl_2_O_4_, CeO_2_, Ce_0.75_Zr_0.25_O_2_, and Co_0.4_Fe_0.6_Al_2_O_4_) were ∼3.6–33 μmol min^−1^ g^−1^ and ∼16.4–102 μmol min^−1^, respectively, at 1000–1350 °C.^10–12^ These reported values are comparable or lower than the results presented here. Typically, ion beam irradiation of an organic material can result in highly reactive free radicals by homolytic dissociation reactions, because organic materials are ionized and excited by the kinetic energy lost by ions colliding with the target materials.^13–15^ The determined activation energy of the 3D microporous NiO_*x*_ structure was ∼1456 kJ mol^−1^. The proton irradiation method with PVP proposed in this study induces dissociation reaction of PVP by the physical and chemical energy of the protons to generate free radicals. Since the reduction of NiO_*x*_ is promoted by highly reactive free radicals, the reduction efficiency of NiO_*x*_ with PVP is higher than that of NiO_*x*_ without PVP, resulting in an increase in hydrogen generation.

Fig. 2(b) shows the hydrogen generation and hydrogen regeneration characteristics of the 3D microporous NiO_*x*_ structure. At 800 °C, the surface of the 3D microporous NiO_*x*_ structure reacted with water vapor and oxidized, and hydrogen generation rapidly increased to a maximum within the initial 20 min. As the oxidation gradually becomes saturated, the hydrogen generation decreased to ∼40% of the maximum after ∼250 min. In the repetitive hydrogen regeneration experiment, the rate was similar to the initial maximum rate of ∼4.2 μmol min^−1^ g^−1^. Fig. 2(c) shows the degradation rates (*C*/*C*_0_) for hydrogen generation during repetitive redox cycles by proton irradiation of the 3D microporous NiO_*x*_ structure, where *C*_0_ refers to the maximum amount of hydrogen and *C* refers to the amount of hydrogen at a certain time. As shown in Fig. 2(c), a similar *C*/*C*_0_ value during hydrogen regeneration confirms that the reduction method of proton irradiation with PVP for hydrogen regeneration is stable.

**Fig. 3:**
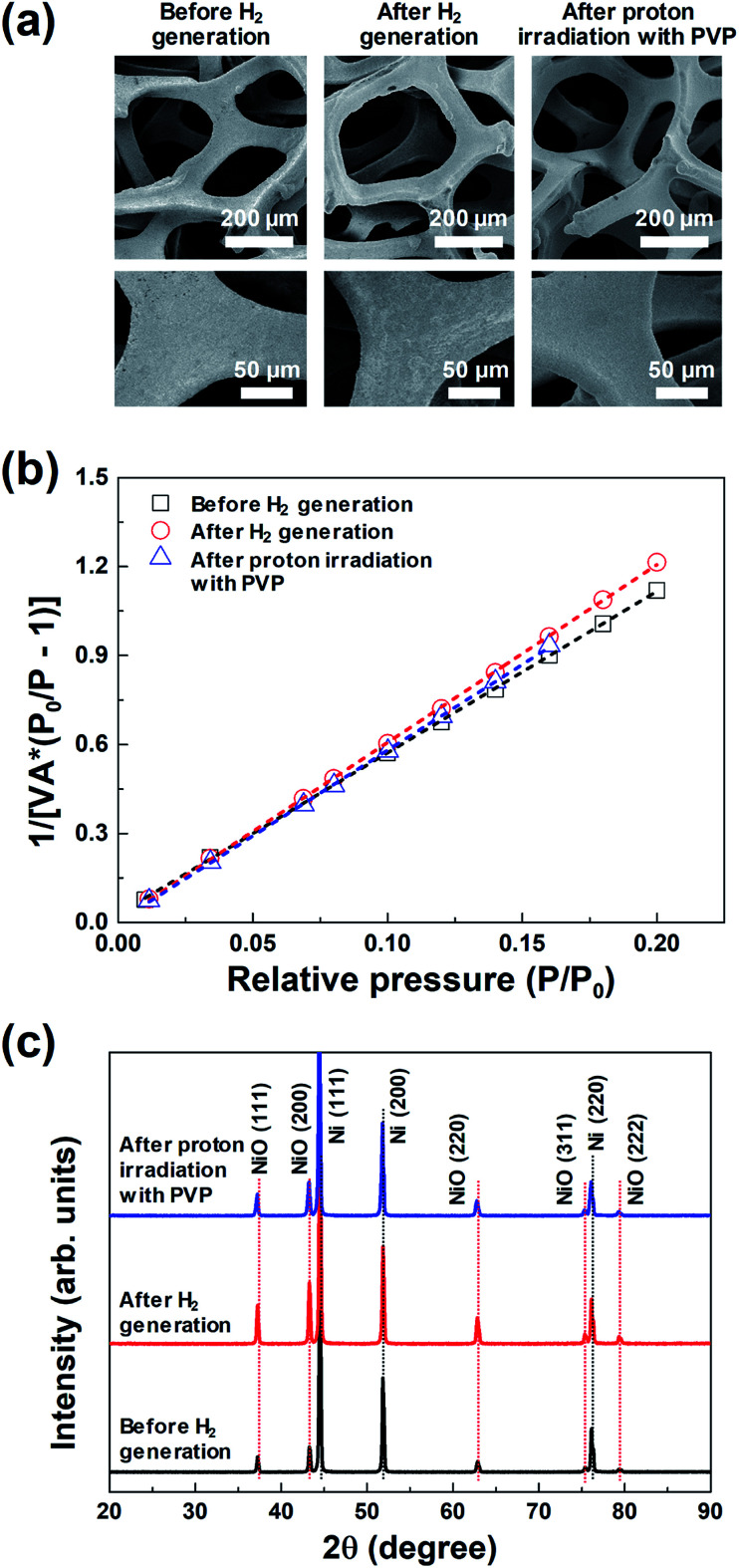
(a) FE-SEM images, (b) BET surface area plots, and (c) XRD profiles of the same 3D microporous NiO_*x*_ structure before and after hydrogen generation, and after proton irradiation with PVP.

Typically, in the case of thermochemical cycles with metal oxide catalysts, the amount of Ni to be oxidized is proportional to the amount of hydrogen generated, as water splitting occurs during the conversion of metal into metal oxide. To identify the ratio of NiO_*x*_ produced by the reaction of Ni and H_2_O vapor, TGA was performed on the samples after initial hydrogen generation as well as on samples undergoing redox reaction after proton irradiation with PVP (hydrogen regeneration). As seen in Fig. 2(d), for the pristine samples before the initial hydrogen generation, the oxidation of Ni to NiO_*x*_ began at approximately 500 °C, and about 0.10, 0.47, and 1.35% NiO_*x*_ (wt%) of were produced at approximately 600, 700, and 800 °C, respectively. Furthermore, the samples reduced by proton irradiation with PVP began to oxidize Ni at temperatures slightly higher than 500 °C with approximately 0.06, 0.34, and 1.42% NiO_*x*_ (wt%) produced at about 600, 700, and 800 °C, respectively. On the other hand, oxidation of Ni in the proton irradiation only samples began at approximately 600 °C, with about 0.02, 0.15, and 0.60% NiO_*x*_ (wt%) produced at approximately 600, 700, and 800 °C, respectively. A comparison of the amounts of NiO_*x*_ confirms that complete conversion occurs in the sample subjected to redox reaction by proton irradiation with PVP after hydrogen generation. On the other hand, ∼50% conversion was observed for the sample proton irradiated without PVP. The conversion rates of hydrogen generation with PVP or without PVP are consistent with the amounts of generated hydrogen, as evident in Fig. 3(a). The proton irradiation method used for the reduction process reduces the oxygen-rich Ni surface and increases the amount of oxygen vacancies (
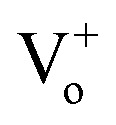
 and 
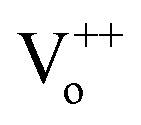
, respectively) on the NiO_*x*_ surface, further reacting with H_2_O vapor to produce hydrogen by the hydrogen generation process. Typically, metal oxide-based thermochemical cycles require high thermal energy through direct heating or concentration of sunlight to regenerate the oxygen vacancies in the metal oxide materials.^7,16,17^ However, the proton irradiation reduction method for the oxidized metal oxide has the advantage of room-temperature processing instead of a high temperature condition.

Fig. 3(a) shows the representative FE-SEM images of the 3D microporous NiO_*x*_ structure before and after hydrogen generation, and after proton irradiation with PVP. The pore size and thickness of the 3D microporous NiO_*x*_ structures used for hydrogen generation were ∼230 μm and ∼1.6 mm, respectively. As shown in Fig. 3(a), no change was observed in the surface structure of the 3D microporous NiO_*x*_ structure, which is composed of 3D multi-pore structures. The most important factor that can increase the hydrogen generation efficiency of the hydrogen generation system using the metal oxide is the reaction surface area between the metal oxide material and H_2_O. To enlarge the reaction surface area, a nanoporous structure or nanoparticles are created by surface treatment of the metal oxide material to enhance the reactivity.^18–21^ However, these structures aggregate with each other as the hydrogen generation reaction proceeds, thereby reducing the reaction surface area and inhibiting the gas flow, consequently lowering the reaction rate. The 3D microporous NiO_*x*_ structure proposed in this study is advantageous in that the structure can maintain its shape during the hydrogen generation and regeneration processes because it is composed of a 3D wire mesh structure.

The changes in surface area of the 3D microporous NiO_*x*_ structure by thermochemical water splitting reaction during hydrogen generation were confirmed through the BET method. Fig. 3(b) shows the BET surface area plot of the 3D microporous NiO_*x*_ structure before and after hydrogen generation, and after proton irradiation with PVP, respectively. The measured BET areas in all the three states confirmed that the surface areas were constant at 0.79 (before hydrogen generation), 0.73 (after hydrogen generation), and 0.75 m^2^ g^−1^ (after proton irradiation with PVP). This result indicates that the surface area of the 3D microporous NiO_*x*_ structure is not critically affected by the repetitive process of hydrogen generation and proton irradiation with PVP.

Fig. 3(c) shows the XRD profiles of the 3D microporous NiO_*x*_ structure before and after hydrogen generation, and after proton irradiation with PVP. For all the three conditions, three strong diffraction peaks (2*θ*) appearing at 44.5°, 51.8°, and 76.1° were ascribed to the (111), (200), and (220) planes of the 3D microporous Ni structure, respectively (ICDD PDF Card 87-0712, JCPDS. 2001). Five weak Bragg peaks (2*θ*) were observed at 37.4°, 43.4°, 62.9°, 75.4°, and 79.4°, which corresponded to the (111), (200), (220), (311), and (222) crystal planes (“Bunsenite” phase) of cubic NiO (JCPDS card No. 73-1523), respectively.^22–24^ The changes in the main diffraction peaks for the three conditions showed that the crystal peaks of the cubic NiO increased in intensity after hydrogen generation, while the corresponding NiO peaks decreased in intensity after proton irradiation with PVP. These results indicate that NiO was oxidized by reacting with H_2_O molecules according to the hydrogen generation process, and that it was effectively reduced after proton irradiation with PVP.

**Fig. 4:**
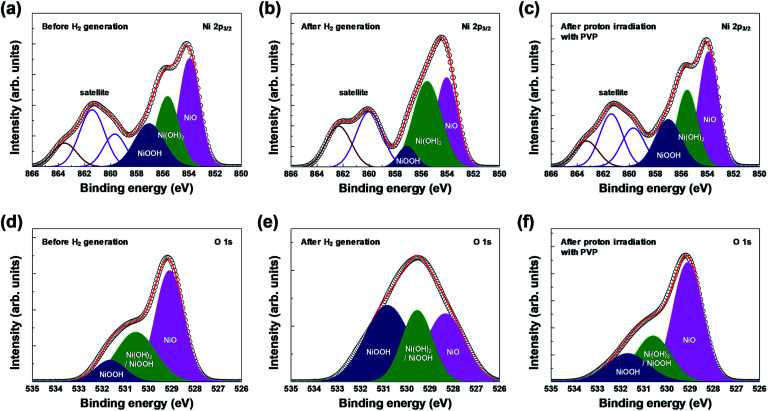
XPS spectra of the 3D microporous NiO_*x*_ structure before and after hydrogen generation, and after proton irradiation with PVP: (a–c) Ni 2p_3/2_ and (d–f) O 1s regions.

XPS analysis confirmed the chemical composition and chemical states of the outermost layer on the surface of the 3D microporous NiO_*x*_ structure. Fig. 4 shows the XPS spectra of Ni 2p_3/2_ and O 1s before and after hydrogen generation, and after proton irradiation with PVP. As shown in Fig. 4(a) and (d), the XPS spectra for each condition showed Ni^2+^ and Ni^3+^ peaks related to the three oxidation states, NiO (854.0 eV for Ni 2p_3/2_, 529.1 eV for O 1s), Ni(OH)_2_ (855.6 eV for Ni 2p_3/2_, 530.6 eV for O 1s), and NiOOH (857.1 eV for Ni 2p_3/2_, 530.6 and 531.7 eV for O 1s).^25–29^ Typically, Ni(OH)_2_ and NiOOH peaks are formed by exposure to ambient humidity or by absorption of oxygen.^30^ Furthermore, the broad peak at ∼861 eV for Ni 2p_3/2_ is related to a “shake-up” satellite due to Ni 3d → Ni 4s transition, which is known to occur by excitation to higher energy levels through interactions between the valence electrons and outgoing electrons.^31,32^ The changes in oxidation before and after hydrogen generation and after proton irradiation with PVP were identified by calculating the ratio of Ni(OH)_2_ (855.6 eV) and NiOOH (857.1 eV) peak areas to the NiO (854.0 eV) peak area in Ni 2p_3/2_ and O 1s states. NiO was oxidized into Ni(OH)_2_ and NiOOH, and the (Ni(OH)_2_ and NiOOH)/NiO peak area increased from ∼1.29 (Ni 2p_3/2_)/∼0.84 (O 1s) to ∼1.47 (Ni 2p_3/2_)/∼2.10 (O 1s), respectively. However, the peak area decreased to 1.27 (Ni 2p_3/2_) and ∼0.78 (O 1s) due to the reduction effect after proton irradiation with PVP.

## 4. Conclusions

In summary, we investigated a method that can easily reduce NiO_*x*_-based reaction medium used for hydrogen generation in the thermochemical water-splitting cycle at room temperature rather than through conventional high-temperature heating. Proton irradiation using PVP as a reducing catalyst proposed in this study allowed further generation of abundant oxygen vacancies because the free radicals generated by the physical and chemical energy of the proton easily release oxygen from the oxygen-rich 3D microporous NiO_*x*_ structure after hydrogen generation. Furthermore, because the metal oxide is reduced at room temperature by proton irradiation, the lowering of reactivity of the metal oxide due to redox reaction is prevented owing to the sintering of metal oxide, and ∼4.2 μmol min^−1^ g^−1^ of hydrogen could be produced at 800 °C even in the repetitive thermochemical cycle, which is similar to the amount generated during initial hydrogen generation. The introduction of the oxygen reduction process using proton irradiation with PVP instead of the conventional reduction can solve the inefficiency issue that the energy required for hydrogen generation is higher than the energy of the generated hydrogen.

## Conflicts of interest

There are no conflicts to declare.

## Supplementary Material
